# Super fine cerium hydroxide abrasives for SiO_2_ film chemical mechanical planarization performing scratch free

**DOI:** 10.1038/s41598-021-97122-9

**Published:** 2021-09-06

**Authors:** Young-Hye Son, Gi-Ppeum Jeong, Pil-Su Kim, Man-Hyup Han, Seong-Wan Hong, Jae-Young Bae, Sung-In Kim, Jin-Hyung Park, Jea-Gun Park

**Affiliations:** 1 Department of Nanoscale Semiconductor Engineering, Hanyang University, Seoul, 04763 Republic of Korea; 2 Department of Electronics and Communications Engineering, Hanyang University, Seoul, 04763 Republic of Korea; 3 Department of Energy Engineering, Hanyang University, Seoul, 04763 Republic of Korea; 4 UB Materials Inc., Yongin, Gyeonggi-do 17162 Republic of Korea

**Keywords:** Chemistry, Engineering, Materials science, Nanoscience and technology

## Abstract

Face-centered-cubic crystallized super-fine (~ 2 nm in size) wet-ceria-abrasives are synthesized using a novel wet precipitation process that comprises a Ce^4+^ precursor, C_3_H_4_N_2_ catalyst, and NaOH titrant for a synthesized termination process at temperature of at temperature of 25 °C. This process overcomes the limitations of chemical–mechanical-planarization (CMP)-induced scratches from conventional dry ceria abrasives with irregular surfaces or wet ceria abrasives with crystalline facets in nanoscale semiconductor devices. The chemical composition of super-fine wet ceria abrasives depends on the synthesis termination pH, that is, Ce(OH)_4_ abrasives at a pH of 4.0–5.0 and a mixture of CeO_2_ and Ce(OH)_4_ abrasives at a pH of 5.5–6.5. The Ce(OH)_4_ abrasives demonstrate better abrasive stability in the SiO_2_-film CMP slurry than the CeO_2_ abrasives and produce a minimum abrasive zeta potential (~ 12 mV) and a minimum secondary abrasive size (~ 130 nm) at the synthesis termination pH of 5.0. Additionally, the abrasive stability of the SiO_2_-film CMP slurry that includes super-fine wet ceria abrasives is notably sensitive to the CMP slurry pH; the best abrasive stability (i.e., a minimum secondary abrasive size of ~ 130 nm) is observed at a specific pH (6.0). As a result, a maximum SiO_2_-film polishing rate (~ 524 nm/min) is achieved at pH 6.0, and the surface is free of stick-and-slip type scratches.

## Introduction

Recently, dynamic random-access memory (DRAM) has been scaled down to ~ 1 nm per year, and NAND-flash memory has been stacked with ~ floors per two years^1–8^. Additionally, logic devices for application processors (APs), central processing units (CPUs), and graphic processing units (GPUs) have been scaled down from 7 nm to 5, 3, and 2 nm, respectively^9–11^. The scaling-down speed of nanoscale semiconductor devices has slowed down, owing to the lithography limit as a result of large surface topography and device structure complexity^12–17^. In particular, the need for chemical mechanical planarization (CMP) for the removal of such surface topography has increased in nanoscale semiconductor device fabrication^18–21^. Among the CMP processes, shallow trench isolation (STI) CMP is an essential fabrication process for all nanoscale semiconductor devices, and poly-Si stop CMP is also an inevitable fabrication process for three-dimensional (3D) NAND-flash memory^22–26^.

**Figure 1:**
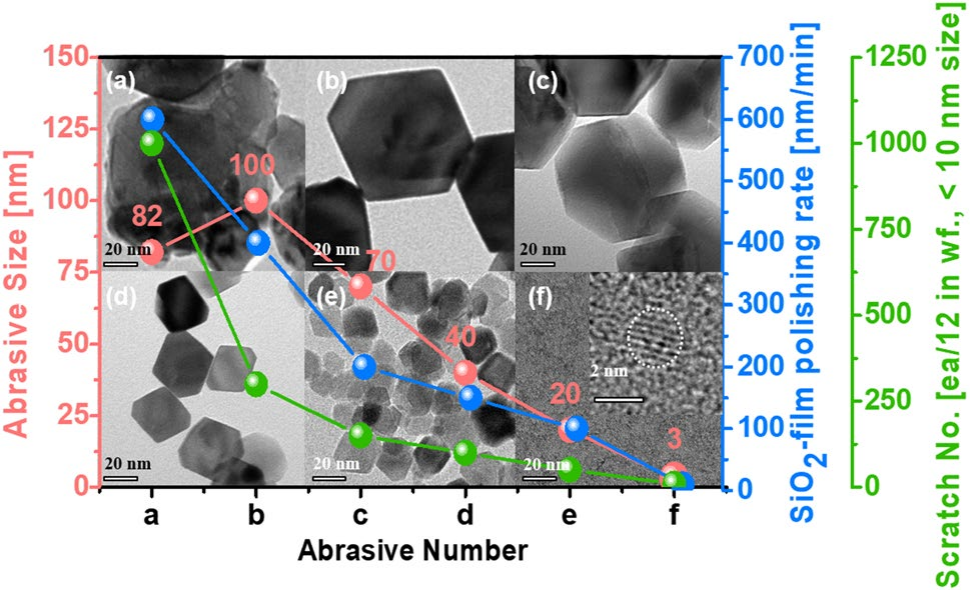
Dependencies of the SiO_2_ film polishing rate and allowable scratch number on the size of dry or wet CeO_2_ abrasives. (**a**) 82 nm dry CeO_2_ abrasives, (**b**) 100 nm, (**c**) 70 nm, (**d**) 40 nm, (**e**) 20 nm, and (**f**) 3 nm wet CeO_2_ abrasives. Dry CeO_2_ abrasives with an irregular surface are produced via a milling process of cerium carbonate, whereas wet ceria abrasives containing f.c.c.-crystalline facets are synthesized by a conventional wet precipitation process at 60–80 °C.

Historically, both STI and poly-Si stop CMP have been performed using a nanoscale (~ 100 nm in size) crystalline cerium oxide abrasive (CeO_2_)-based CMP slurry. In particular, the surface morphology of crystalline cerium oxide abrasives has recently changed from a sharp surface (i.e., dry cerium oxide abrasives) to a crystalline-facet surface (i.e., wet cerium oxide abrasives) with {100}, {110}, and {111} to minimize the CMP-induced scratches produced by the stick-and-slip mechanism, as shown in Figs. 1a–b and S1. Additionally, the size of wet cerium oxide abrasives with crystalline-facet surfaces has been rapidly reduced because the allowable size of CMP-induced scratches has significantly decreased with the scaling down of nanoscale semiconductor devices^27–31^. However, CMP performance has faced the fundamental issue of a trade-off between the SiO_2_, Si_3_N_4_, or poly-Si film polishing rate and the remaining CMP induced scratches; that is, a smaller wet cerium oxide abrasive size leads to fewer remaining CMP-induced scratches and a lower film polishing rate, as shown in Fig. 1. This trade-off meets the application limit of a wet cerium oxide abrasive-based CMP slurry for the further scaling-down of nanoscale DRAM, 3D NAND-flash, and logic devices.

In this study, we developed crystalline cerium hydroxide (CeOH_4_) abrasives with a size of ~ 2 nm (called super-fine CeOH_4_ abrasives) at temperature of 25 °C. This is an alternative to crystalline cerium oxide (CeO_2_) abrasives, which are produced via wet precipitation synthesis using a Ce^4+^ precursor, catalyst for enhancing Ce^4+^ solubility, and pH titrant. Note that crystalline CeO_2_ abrasives are synthesized using a Ce^4+^ seed precursor and Ce^3+^ growth precursor at 60–80 °C, as shown in Fig. S2. First, we demonstrated in detail the mechanism by which super-fine wet Ce(OH)_4_ abrasives are synthesized via wet precipitation using a Ce^4+^ precursor, catalyst and pH titrant. In addition, we use a CMP slurry with super-fine wet Ce(OH)_4_ abrasives to investigate the dependence of the absorbance of wet cerium hydroxide abrasives, secondary abrasive size, the zeta potential of wet cerium hydroxide abrasives, and CMP performance on the synthesis termination pH and CMP slurry pH.

## Results

### Super-fine wet cerium hydroxide (Ce(OH)_4_)) abrasive synthesis mechanism

**Figure 2:**
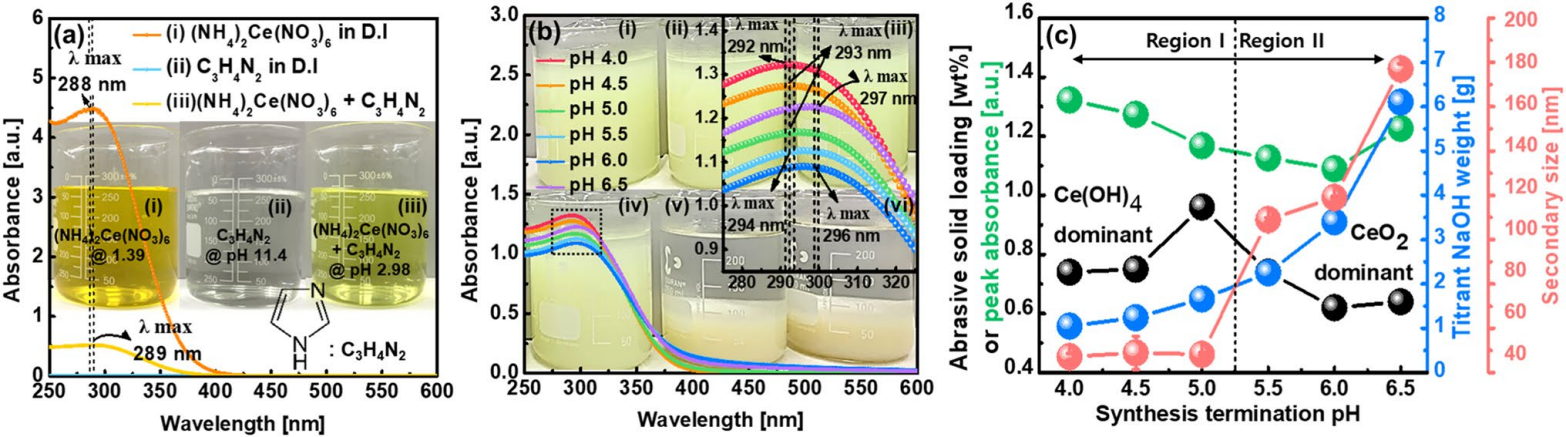
Dependencies of chemical and material properties of super-fine wet ceria abrasives on synthesis termination pH. (**a**) Chemical solution color of (i) precursor (NH_4_)_2_Ce(NO_3_)_6_ dissolved in DI water, (ii) C_3_H_4_N_2_ dissolved in DI water, and (iii) (NH_4_)_2_Ce(NO_3_)_6_ and C_3_H_4_N_2_ dissolved in DI water. (**b**) Peak absorption wavelength and absorbance dependent on synthesis termination pH. Beaker (i), (ii), (iii), (iv), (v), and (vi) of the D.I. solutions with dispersed super-fine wet ceria abrasives were terminated at a pH of 4.0, 4.5, 5.0, 5.5, 6.0, and 6.5, respectively, following a synthesis. (**c**) Titrant NaOH weight, abrasive solid loading, peak absorbance, and secondary abrasives size, dependent on synthesis termination pH.

Nanoscale (5–100 nm in size) wet ceria abrasives were typically synthesized using Ce^3+^ and Ce^4+^ precursors at > 60 °C, as shown in Fig. S2. However, super-fine (~ 2 nm in size) wet ceria abrasives were synthesized using only the Ce^4+^ precursor at temperature of 25 °C. The synthesis mechanism of super-fine wet ceria abrasives can be analyzed by observing the color of the abrasives in a solution after synthesis. Ammonium cerium nitrate ((NH_4_)_2_Ce(NO_3_)_6_)) was utilized as a Ce^4+^ precursor and imidazole (C_3_H_4_N_2_) as a catalyst to enhance the Ce^4+^ solubility. Both were dissolved in deionized (DI) water; the dissolved (NH_4_)_2_Ce(NO_3_)_6_ exhibited a transparent orange color, and C_3_H_4_N_2_ was colorless, as shown in beakers (i) and (ii) of Fig. 2a. After mixing (NH_4_)_2_Ce(NO_3_)_6_ with C_3_H_4_N_2_, the mixed chemical presented a transparent and bright yellow color, as shown in beaker (iii) of Fig. 2a. The absorbance of the (NH_4_)_2_Ce(NO_3_)_6_ solution (at pH 1.39) and the mixed (NH_4_)_2_Ce(NO_3_)_6_ solution (at pH 2.98) and C_3_H_4_N_2_ solution (at pH 11.40) peaked at wavelengths of 288 nm and 289 nm, respectively. Note that the C_3_H_4_N_2_ chemical was added to the (NH_4_)_2_Ce(NO_3_)_6_ solution at a flow rate of 10 ml/min, and the two chemicals were mixed via stirring. Then, sodium hydroxide (NaOH) was added to titrate the pH of the mixed chemical (i.e., 4.0, 4.5, 5.0, 5.5, 6.0, and 6.5), which is called a synthesis termination process. The color of the solution containing abrasives after the synthesis termination process was dependent on the termination pH, as shown in the beakers in Fig. 2b. It changed from opaque and bright yellow (beaker (i) of Fig. 2b) to pale yellow (beaker (vi) of Fig. 2b) when the synthesis termination pH was adjusted from 4.0 to 6.5. In particular, at a pH above 6.0, the abrasives in the solution were completely sediment. To investigate the detailed color of the synthesized abrasives, the solutions including the abrasives were centrifuged three times and dispersed in DI water; the pH values were between 3.10–3.21. At a pH of 4.0, the synthesized abrasives were bright yellow, as shown in the centrifugal bottle of Fig. S3a. This is the typical color of Ce(OH)_4_ powder, as shown in Fig. S4a, which indicates that the chemical composition of the synthesized abrasives at a pH of 4.0 is Ce(OH)_4_. However, at a pH 6.5, the abrasives exhibited a pale yellow color, as shown in the centrifugal bottle in Fig. S3f, which is the typical color of CeO_2_ powder, as shown in Fig. S4b. This indicates that the chemical composition of the synthesized abrasives (pH 6.5) was CeO_2_. In addition, from a pH of 4.5 to 6.0, the color of the synthesized abrasives changed from bright yellow to pale yellow, which implies that their chemical composition is a mixture of Ce(OH)_4_ and CeO_2_, as shown in the centrifugal bottle in Fig. S3b–e. In particular, the chemical composition of the synthesized abrasives at a pH of 6.0 would be almost CeO_2_. These results indicate that the color of the synthesized abrasives (i.e., the chemical composition of super-fine wet ceria abrasives) is dominantly dependent on the synthesis termination process pH. The difference between CeO_2_ and Ce(OH)_4_ can be recognized by analyzing the chemical composition between them using XPS, as shown in Fig. S5. The relative XPS peak signals of CeO_2_, Ce(OH)_4_ and Ce_2_O_3_ are found at 529.29, 530.79, and 532.29 eV, respectively. We prepare 3 samples such as commercial CeO_2_ (UB materials Co.), commercial Ce(OH)_4_ (Sigma Aldrich Co.), and Ce(OH)_4_ (our synthesis) abrasives. The sequence of a higher relative XPS intensity of CeO_2_ was followed by commercial CeO_2_, commercial Ce(OH)_4_, and Ce(OH)_4_ abrasives. Otherwise, the order of a higher relative XPS intensity of Ce(OH)_x_ was followed by Ce(OH)_4,_ commercial Ce(OH)_4_, and commercial CeO_2_ abrasives. Thus, these results indicate that the chemical composition of Ce(OH)_4_ is different from that of CeO_2._ From a pH of 4.0 to 5.0 (region I), the main synthesized abrasives are the Ce(OH)_4_ abrasives; however, from a pH of 5.5 to 6.5 (region II), a mixture of the Ce(OH)_4_ and CeO_2_ abrasives are evident. In particular, the synthesized abrasives at a pH of 6.5 are principally CeO_2_ abrasives.

**Figure 3:**
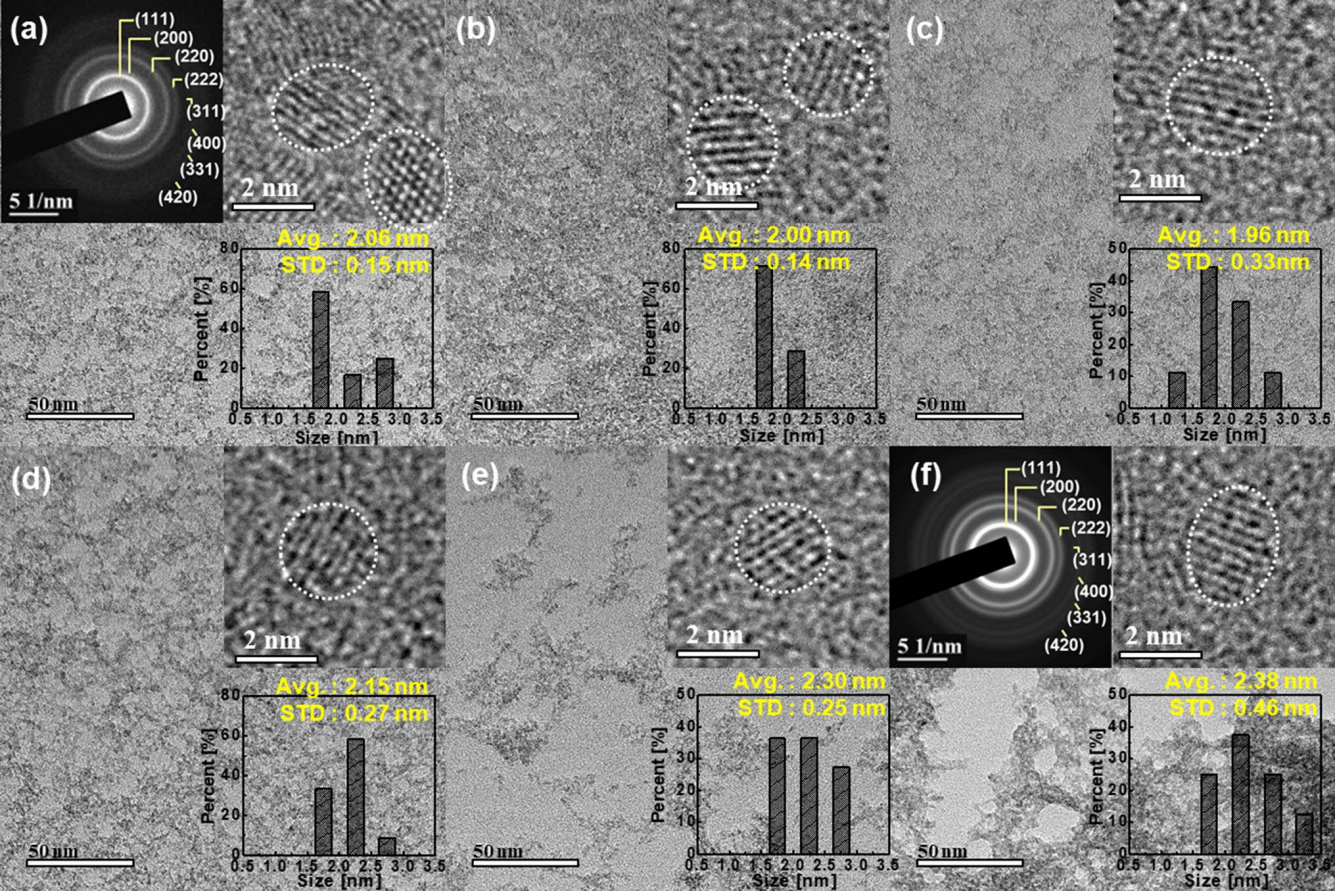
High-resolution TEM images of super-fine wet ceria abrasives with synthesis termination pH values of (**a**) 4.0, (**b**) 4.5, (**c**) 5.0, (**d**) 5.5, (**e**) 6.0, and (**f**) 6.5. The background TEM images were obtained at a 50-nm scale (lower left), whereas the TEM images in the insets of the figures (upper right) were observed at a 2-nm scale. In addition, a *μ*-crystalline diffraction pattern of super-fine wet ceria abrasives were presented in the inset of the figures (upper left), shown in Fig. S6.

The dependence of the titrant (NaOH) weight and abrasive solid loading after synthesis on the synthesis termination pH were investigated to estimate the dispersion ability of super-fine wet ceria abrasives in a DI solution. The NaOH weight exponentially increased with the synthesis termination pH, which is a typical titration method; it increased from 1.05 to 6.01 g when the pH increased from 4.0 to 6.5, as shown in Fig. 2c. Additionally, the abrasive solid loading weight after synthesis (~ 0.96 wt%) peaked at a pH of 5.0. Between a pH of 4.0 and 5.0 (region I), the abrasive solid loading weight considerably increased from 0.74 to 0.96 wt%; therefore, the Ce(OH)_4_ growth rate increased with the synthesis termination pH because it was enhanced with the titrant NaOH weight. Hence, the Ce(OH)_4_ abrasive color in all solutions was bright yellow, as shown in beakers (i)–(iii) of Fig. 2b, because the Ce(OH)_4_ abrasives solid loading weight increased with the synthesis termination pH. However, between a pH of 5.5 and 6.5 (region II), the abrasive solid loading weight significantly decreased from 0.74 to 0.64 wt%. This implies that the CeO_2_ abrasive growth rate in the solution is higher than that of Ce(OH)_4_ because the CeO_2_ abrasives and H_2_O molecules are produced via the reaction of the Ce(OH)_4_ abrasives with OH^−^ in the solution. It should be noted that the CeO_2_ molecular weight (172 g/mol) is lighter than that of Ce(OH)_4_ (208 g/mol). Thus, the abrasive solid loading weight decreased as the synthesis termination process pH increased. As a result, the abrasive color varied from dark bright yellow to pale yellow, as shown in beakers (iv)–(vi) of Figs. 3b, S3d–f. The dependency of the abrasive solid loading after synthesis on the synthesis termination pH indicates that the abrasive solid loading weight (~ 0.96 wt%) peaked at a specific synthesis termination pH (i.e., pH 5.0) because a maximum amount of Ce(OH)_4_ abrasives was produced at a pH of 5.0.

The dependency of the abrasive solid loading weight after three centrifugation cycles on the synthesis termination pH was confirmed by observing the dependencies of the peak absorbance wavelength and the peak absorbance in the DI solution that included abrasives on the synthesis termination pH. The peak absorbance wavelength increased continuously from 292 to 297 nm when the synthesis termination pH increased from 4.0 to 6.5, as shown in the inset of Fig. 2b. This indicates that the chemical composition of the abrasives transformed from Ce(OH)_4_ to CeO_2_ and is well correlated with the dependency of the abrasive color on the synthesis termination pH in beakers (i)–(vi) of Fig. 2b. Note that the peak absorbance wavelengths of the Ce(OH)_4_ and CeO_2_ abrasives were at ~ 293 and ~ 297 nm, respectively^32–34^. Moreover, the peak absorbance decreased linearly from 1.325 to 1.090 a.u. when the synthesis termination pH increased from 4.0 to 6.0; it then abruptly increased to 1.226 a.u. at the synthesis termination pH of 6.5, which is likely related to the increase in the secondary abrasive size dispersed in a DI solution. Furthermore, the secondary abrasive size in a DI water solution was sustained at ~ 48 nm between the synthesis termination pH values of 4.0 and 5.0 (region I), which indicates that the super-fine abrasives were well dispersed, as shown Fig. 2c. For a further pH increase from 5.5 to 6.5, the secondary abrasive size exponentially increased with the synthesis termination pH; thus, the abrasive dispersion ability decreased at the synthesis termination pH of 5.5–6.5 (region II). The dependence of the abrasive secondary size on the synthesis termination pH implies that the Ce(OH)_4_ abrasives in the DI water solution were well dispersed between the termination pH values of 4.0 and 5.0 (region I). However, the CeO_2_ abrasives dispersed in the solution with a pH above 5.5 (region II) became further agglomerated as the pH increased. The absorbance of a solution that contains abrasives generally depends on the dispersant degree of the abrasives in the solution; that is, a higher abrasive dispersant degree in a solution results in a lower abrasive peak absorbance. Thus, the dependency of the peak absorbance in a solution that includes abrasives on the synthesis termination pH is associated with the dependencies of the chemical composition of the synthesized abrasives and the secondary abrasive size on the synthesis termination pH.

The morphology of the Ce(OH)_4_ or CeO_2_ abrasives dependent on the synthesis termination pH was observed using high-resolution(HR) transmission electron microscopy (TEM). At a pH of 4.0, the Ce(OH)_4_ abrasives were well crystallized, and the average and standard deviation of their size were 2.06 and 0.15 nm, respectively; these are referred to as super-fine wet ceria abrasives, as shown in the HR-TEM image in Fig. 3a. In addition, their *u*-diffraction pattern exhibited a typical polycrystalline diffraction pattern with a higher intensity sequence of (111), (220), (311), (200), (400), (331), and (420), as shown in the *u*-diffraction pattern of Fig. 3a. This diffraction pattern was consistent with the crystalline planes and higher intensity sequence of crystalline wet ceria abrasives produced by a typical wet precipitation method at 60–80 °C, as shown in Fig. S2. Up to a pH of 5.0, the size and crystalline planes of the Ce(OH)_4_ abrasives appeared constant, as shown in Figs. 3a–c and S6a–c. The size of the mixture of the Ce(OH)_4_ and CeO_2_ abrasives increased very slightly with the synthesis termination pH; however, their polycrystalline planes were similar to that of the Ce(OH)_4_ abrasives, as shown in Figs. 3d–f and S6d–f. Therefore, super-fine wet ceria abrasives ~ 2 nm in size were synthesized at temperature of 25 °C using only a Ce^4+^ precursor.

To understand whether Ce(OH)_4_ or CeO_2_ abrasives were synthesized, the chemical reaction equations of super-fine wet ceria abrasives are reviewed in detail as follows:

1 $$4\left( {{\text{NH}}_{4} } \right)_{2} {\text{Ce}}\left( {{\text{NO}}_{3} } \right)_{6} + {\text{H}}_{2} {\text{O}} \to 4\left[ {{\text{Ce}}\left( {{\text{NO}}_{3} } \right)_{6} } \right]^{2 - } + 8{\text{NH}}^{4 + } + {\text{OH}}^{ - } + {\text{H}}^{ + }$$

2 $$\begin{aligned} & 4\left[ {{\text{Ce}}\left( {{\text{NO}}_{3} } \right)_{6} } \right]^{2 - } + 8{\text{NH}}^{4 + } + {\text{OH}}^{ - } + {\text{H}}^{ + } + {\text{C}}_{3} {\text{H}}_{4} {\text{N}}_{2} \\ & \quad \to 4{\text{Ce}}^{4 + } + 24{\text{NO}}^{3 - } + 8{\text{NH}}^{4 + } + {\text{OH}}^{ - } + {\text{C}}_{3} {\text{H}}_{3} {\text{N}}_{2}^{ - } + 2{\text{H}}^{ + } \\ \end{aligned}$$

3 $$\begin{aligned} & 4{\text{Ce}}^{4 + } + {\text{C}}_{3} {\text{H}}_{3} {\text{N}}_{2}^{ - } + 2{\text{H}}^{ + } + 11{\text{Na}}^{ + } + 12{\text{OH}}^{ - } \\ & \quad \to 3{\text{Ce}}\left( {{\text{OH}}} \right)_{4} + {\text{Ce}}^{4 + } + {\text{NaC}}_{3} {\text{H}}_{3} {\text{N}}_{2} + 10{\text{Na}}^{ + } + 2{\text{H}}^{ + } \\ \end{aligned}$$

4 $$\begin{aligned} & 4{\text{Ce}}^{4 + } + {\text{C}}_{3} {\text{H}}_{3} {\text{N}}_{2}^{ - } + 2{\text{H}} + 13{\text{Na}}^{ + } + 14{\text{OH}}^{ - } \\ & \quad \to 3{\text{Ce}}\left( {{\text{OH}}} \right)_{4} + {\text{Ce}}\left( {{\text{OH}}} \right)_{2}^{2 + } + {\text{NaC}}_{3} {\text{H}}_{3} {\text{N}}_{2} + 12{\text{Na}}^{ + } + 2{\text{H}}^{ + } \\ \end{aligned}$$

5 $$4{\text{Ce}}^{4 + } + {\text{C}}_{3} {\text{H}}_{3} {\text{N}}_{2}^{ - } + 2{\text{H}} + 15{\text{Na}}^{ + } + 16{\text{OH}}^{ - } \to 4{\text{Ce}}\left( {{\text{OH}}} \right)_{4} + {\text{NaC}}_{3} {\text{H}}_{3} {\text{N}}_{2} + 14{\text{Na}}^{ + } + 2{\text{H}}^{ + }$$

6 $$\begin{aligned} & 4{\text{Ce}}^{4 + } + {\text{C}}_{3} {\text{H}}_{3} {\text{N}}_{2}^{ - } + 2{\text{H}}^{ + } + 19{\text{Na}}^{ + } + 20{\text{OH}}^{ - } (access\;NaOH) \\ & \quad \to 3{\text{Ce}}\left( {{\text{OH}}} \right)_{4} + {\text{CeO}}_{2} + 4{\text{H}}_{2} {\text{O}} + {\text{O}}_{2} + {\text{NaC}}_{3} {\text{H}}_{3} {\text{N}}_{2} + 18{\text{Na}}^{ + } \\ \end{aligned}$$

7 $$\begin{aligned} & 4{\text{Ce}}^{4 + } + {\text{C}}_{3} {\text{H}}_{3} {\text{N}}_{2}^{ - } + 2{\text{H}}^{ + } + 27{\text{Na}}^{ + } + 28{\text{OH}}^{ - } (access\;NaOH) \\ & \quad \to {\text{Ce}}\left( {{\text{OH}}} \right)_{4} + 3{\text{CeO}}_{2} + 14{\text{H}}_{2} {\text{O}} + 6{\text{O}}_{2} + {\text{NaC}}_{3} {\text{H}}_{3} {\text{N}}_{2} + 26{\text{Na}}^{ + } \\ \end{aligned}$$

8 $$\begin{aligned} & 4{\text{Ce}}^{4 + } + {\text{C}}_{3} {\text{H}}_{3} {\text{N}}_{2}^{ - } + 2{\text{H}}^{ + } + 31{\text{Na}}^{ + } + 32{\text{OH}}^{ - } (access\;NaOH) \\ & \quad \to 4{\text{CeO}}_{2} + 16{\text{H}}_{2} {\text{O}} + 4{\text{O}}_{2} + {\text{NaC}}_{3} {\text{H}}_{3} {\text{N}}_{2} + 30{\text{Na}}^{ + } . \\ \end{aligned}$$

The dissolution of ammonium cerium nitrate (i.e., (NH_4_)_2_Ce(NO_3_)_6_) as a precursor to DI water produces [Ce_9_NO_3_)_6_]^2−^, NH^4+^, OH^−^, and H^+^, which exhibits a transparent orange color, as shown in Eq. (1) and (i) in Fig. 2a. The addition of imidazole (C_3_H_4_N_2_) into the solution in Eq. (1) as a catalyst enhances the solubility of Ce^4+^, and the solution then exhibits a transparent and bright yellow color, as shown in Eq. (2) and beaker (iii) of Fig. 2a. Between pH values of 4.0 and 5.0 (region I), the addition of NaOH as a synthesis termination titrant produces the Ce(OH)_4_ abrasives, and a higher synthesis termination pH (i.e., NaOH titrant amount) leads to a higher solid loading weight of the Ce(OH)_4_ abrasives. Additionally, the synthesized abrasives exhibited a bright yellow color, as shown in Eq. (3)–(5), beakers (i) and (iii) of Figs. 2b, and S3a–c. However, between the pH values of 5.5 and 6.0, further addition of the NaOH titrant generates a mixture of Ce(OH)_4_ and CeO_2_ abrasives; thus, a larger amount of NaOH titrant results in more CeO_2_ abrasives than Ce(OH)_4_ abrasives, as shown in Eq. (6) and (7), beakers (iv) and (v) of Figs. 2b, and S3d–e. Moreover, at a pH of 6.5, further addition of the NaOH titrant primarily generates the CeO_2_ abrasives, which are completely sediment after adding the NaOH titrant to the synthesized solution, as shown in Eq. (8), beaker (vi) of Figs. 2b and S3f. Thus, between the pH values of 5.5 and 6.5 (region II), the abrasive solid loading concentration significantly decreased with increasing synthesis termination pH, and the abrasive color changed from dark yellow to pale yellow and dark pale yellow, as shown in Figs. 2c, S3d–f. The chemical reaction equations of the super-fine wet ceria abrasive synthesis dependent on the synthesized pH clearly explain the synthesis mechanism of super-fine (~ 2 nm in size) wet ceria abrasives, and the precise chemical composition of super-fine wet ceria abrasives are extremely sensitive to the synthesis termination pH.

### Dependency of SiO_2_-film polishing rate on synthesis termination pH of super-fine wet ceria abrasives

The synthesized wet ceria abrasives (i.e., facet-surface CeO_2_ abrasives) produced by a conventional wet precipitation method at 60–80 °C generally encounters an extreme limitation regarding the trade-off between the SiO_2_-film polishing rate and the remaining scratches after CMP, which is dependent on the wet ceria abrasive size; that is, a smaller wet ceria abrasive size leads to a lower SiO_2_-film polishing rate and fewer remaining scratches after CMP, as shown in Fig. 1. To achieve minimal stick-and-slip type scratches induced by a SiO_2_-film CMP using conventional facet-surface wet ceria-abrasives, the wet abrasive size must be less than 3 nm. However, its SiO_2_-film polishing rate performed less than 10 nm/min for a 300-mm-diameter SiO_2_-film CMP, which cannot be applied for the CMP process for nanoscale semiconductor devices. Thus, whether or not the super-fine (~ 2 nm in size) wet ceria abrasive (Ce(OH)_4_, mixture of CeO_2_ and Ce(OH)_4_, or CeO_2_ abrasives) CMP slurries can overcome the trade-off hurdle of a conventional facet-surface wet ceria abrasive (i.e., CeO_2_ abrasives) is an essential factor in determining the possibility of applying them for the CMP process of nanoscale semiconductor devices.

**Figure 4:**
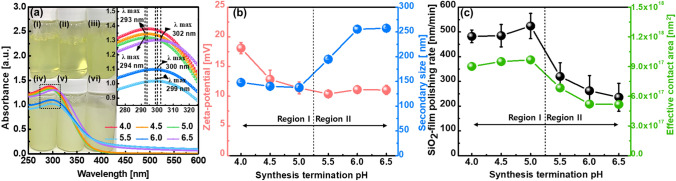
CMP slurry characteristics and CMP performance dependent on synthesis termination pH. (**a**) Peak absorption wavelength and absorbance, (**b**) abrasive zeta potential and secondary abrasives size, and (**c**) SiO_2_-film polishing rate and effective contact area between abrasives and SiO_2_-film surface. In (**a**), the CMP slurries in beakers (i), (ii), (iii), (iv), (v), and (vi) were terminated at pH values of 4.0, 4.5, 5.0, 5.5, 6.0, and 6.5, respectively, following a synthesis.

First, to investigate the dependency of the SiO_2_-film polishing rate on the synthesis termination pH of super-fine wet ceria abrasives, the SiO_2_-film CMP slurries were fabricated using 0.3 wt% super-fine wet ceria abrasives, a dispersant (0.3 wt% polyvinyl alcohol (PVA)), and DI water, a titrant (NaOH) adjusted at a pH of 6.0 of the CMP slurries, as a function of the synthesis termination pH. It was previously mentioned that super-fine wet ceria abrasives were centrifuged three times after synthesis at different termination pH values. Then, all abrasives were again dispersed with PVA and titrated at a pH of 6.0 using NaOH and DI water. The CMP slurry color varied from transparent yellow to opaque yellow when the synthesis termination pH increased from 4.0 to 6.5, as shown in the beakers in Fig. 4a. From a pH of 4.0 to 4.5 (region I), the peak absorbance wavelength of all CMP slurries was ~ 293 nm. However, from a pH of 5.0 to 6.5 (region II), this value increased from 299 to 300 and 302 nm when the synthesis termination pH increased from 5.0 to 5.5 and 6.5, as shown in the inset of Fig. 4a. Additionally, the peak absorbance decreased from 1.397 to 1.099 a.u. when the synthesis termination pH increased from 4.0 to 6.0, and it then abruptly increased to 1.303 at a pH of 6.5. This behavior is similar to the dependency of the peak absorbance of super-fine wet ceria abrasives on the synthesis termination pH in Fig. 2b. In region I, the zeta potential of the super-fine wet ceria abrasive-based CMP slurries rapidly decreased from + 18.1 to + 11.40 mV when the synthesis termination pH increased from 4.0 to 5.0. In region II, it reached ~  + 11.0 mV as the pH increased further, as shown in Fig. 4b. Thus, the abrasive zeta potential exhibited two different regions of dependence on the synthesis termination pH: region I (i.e., zeta potential decrease) and region II (i.e., saturated zeta potential). Moreover, in region I, the secondary abrasive size of the CMP slurries decreased very slightly from 147 to 137 nm when the synthesis termination pH increased from 4.0 to 5.0; however, in region II, it rapidly increased from 195 to 258 nm when the synthesis termination pH increased from 5.5 to 6.5. Hence, the secondary abrasive size also exhibited two different regions of dependence on the synthesis termination pH: region I (i.e., almost constant secondary abrasive size) and region II (i.e., rapid increase in secondary abrasive size). Note that the secondary abrasive size in a CMP slurry responds to the dispersant degree of the primary abrasives (i.e., super-fine wet ceria abrasives), which directly affects the film polishing rate. A larger secondary abrasive in a CMP slurry generally leads to a lower SiO_2_-film polishing rate^35–40^. Furthermore, in region I, the 12-inch-wafer SiO_2_-film polishing rate slightly increased from 482 to 524 nm/min when the synthesis termination pH increased from 4.0 to 5.0. In region II, it rapidly decreased from 524 to 236 nm/min for a further increase in the synthesis termination pH, as shown in Fig. 4c. This result indicates that there were also two different regions of dependence in the 12-inch-wafer SiO_2_-film polishing rate on the synthesis termination pH: region I (i.e., slight SiO_2_-film polishing rate increase) and region II (i.e., rapid SiO_2_-film polishing rate decrease). The region where two regions coincided can be understood by considering three points of view (i.e., abrasive zeta potential, secondary-abrasive size, and effective contact area between abrasives and the SiO_2_-film surface). In region I, the SiO_2_-film polishing rate slightly increased with decreasing abrasive zeta potential and the secondary abrasive size of the CMP slurries because a high attractive static force between the Ce(OH)_4_ abrasives and the SiO_2_-film surface led to a lower SiO_2_-film polishing rate and a larger secondary abrasive size in the CMP slurry, which resulted in a lower SiO_2_-film polishing rate^35–40^. Note that the zeta potentials of the SiO_2_-film surface and Ce(OH)_4_ abrasives are highly negatively charged (~ − 28.8 mV) and positively charged (+ 11.4 to + 18.1 mV), respectively; thus, the static force between the abrasives and the SiO_2_-film surface is a strong attractive force. In region II, the SiO_2_-film polishing rate abruptly decreased above a pH of 5.0 because the secondary abrasive composition transferred from Ce(OH)_4_ at a pH of 5.0 to the mixture of Ce(OH)_4_ at a pH of 5.5; therefore, the secondary abrasive size significantly increased from 137 to 195 nm. Additionally, when the synthesis termination pH increased from 5.5 to 6.5, the SiO_2_-film polishing rate decreased considerably with the synthesis termination pH because the secondary abrasive size in the CMP slurries increased significantly with the termination pH. Furthermore, the dependence of the SiO_2_-film polishing rate on the synthesis termination pH was in good agreement with that of the effective contact area between the abrasives and the SiO_2_-film surface on the synthesis termination pH, as shown in Fig. 4c. Note that the effective contact area between the abrasives and the SiO_2_-film surface was calculated with the assumption that the morphology of super-fine wet ceria secondary abrasives is spherical, and the abrasives are at the fixed abrasive solid loading (0.3 wt%) as a function of the synthesis termination pH. In a SiO_2_-film CMP process, for planarization of the SiO_2_-film surface topography, the secondary-abrasives in a CMP slurry touch and rub the Si(OH)_x_ layer produced by hydrolysis reaction. Hence, the contact area of the secondary-abrasives determines the SiO_2_-film polishing-rate; i.e., a larger total contact-area leads to a higher SiO_2_-film polishing-rate, called contact-area based on CMP mechanism, as shown in Fig. S7. If the solid loading of the abrasives in a SiO_2_-film CMP slurry is fixed with 0.3 wt%, a larger secondary-abrasive size in the CMP slurry would lead to a smaller number of secondary-abrasives in the slurry, indicating that the total contact-area of the CMP slurry having a small secondary-abrasive size would be larger than that having a large secondary-abrasive size. As a result, the SiO_2_-film polishing-rate using the slurry with a small secondary-abrasive size would be higher than that using the slurry with a large secondary-abrasive size. These results prove that the dependence of the SiO_2_-film polishing rate on the synthesis termination pH are related to the abrasive zeta potential, secondary abrasive size, and total abrasive SiO_2_-film-surface contact area. Interestingly, the super-fine (~ 2 nm in size) wet ceria abrasive (Ce(OH)_4_ abrasive)-based CMP slurry achieved minimal stick-and-slip type scratches after the 12-inch-wafer SiO_2_-film CMP, as shown in Fig. S1c. Additionally, the SiO_2_-film polishing rate (~ 524 nm/min) of super-fine (~ 2 nm in size) wet ceria abrasive (Ce(OH)_4_ abrasive)-based CMP slurry was ~ 50 times higher than that (~ 10 nm/min) of the facet surface (~ 3 nm in size) wet ceria abrasive (CeO_2_ abrasive)-based CMP slurry.

### Dependency of SiO_2_-film polishing rate on pH of super-fine wet ceria abrasives-based CMP slurry

**Figure 5:**
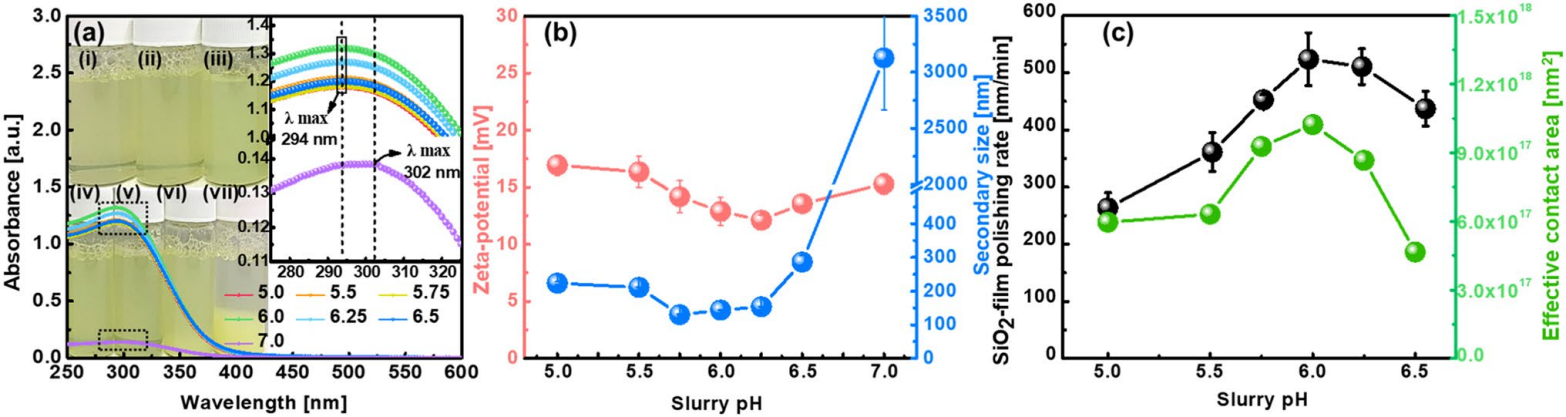
CMP slurry characteristics and CMP performance dependent on pH values of slurries, including super-fine (~ 2 nm in size) Ce(OH)_4_ wet ceria abrasives. (**a**) Peak absorption wavelength and absorbance, (**b**) abrasive zeta potential and secondary abrasives size, and (**c**) SiO_2_-film polishing rate and effective contact area between abrasives and SiO_2_-film surface. In (**a**), the CMP slurries in beakers (i), (ii), (iii), (iv), (v), and (vi) were produced at pH values of 5.0, 5.25, 5.5, 5.75, 6.0, 6.5, and 7.0, respectively, following a synthesis. As previously mentioned, super-fine wet ceria abrasives for all CMP slurries were synthesized at a termination pH of 5.0.

Because the SiO_2_-film CMP slurries that include super-fine wet ceria abrasives (~ 2 nm Ce(OH)_4_ abrasives) exhibited a maximum SiO_2_-film polishing rate at the synthesis termination pH of 5.0, the dependency of the SiO_2_-film polishing rate on the CMP slurry pH was investigated at a solid loading of 0.3 wt% and a dispersant (PVA) concentration of 0.25 wt%. Excluding the CMP slurry with a pH of 7.0, those with pH values between 5.0 and 6.5 exhibited a transparent yellow color, and their abrasives were well dispersed in the slurry, as shown in beakers (i)–(vi) of Fig. 5a. However, the abrasives in the CMP slurry with a pH of 7.0 were completely sediment. Moreover, excluding the CMP slurry with a pH of 7.0, the absorbance of the CMP slurry with pH values between 5.0 and 6.5 peaked at a wavelength of 294 nm. However, the peak absorbance of the CMP slurry at a pH of 7.0 was identified at a wavelength of 302 nm, owing to the sedimentation of abrasives in the CMP slurry. The peak absorbance of the CMP slurries slightly increased with the CMP slurry pH at 5.0–6.0; however, it considerably decreased with increasing CMP slurry pH at 6.25–7.0, as shown in the inset of Fig. 5a. This is similar to the dependence of the peak absorbance on the synthesis termination pH in the inset of Fig. 4a. The abrasive zeta potential of the CMP slurry decreased slightly from + 16.9 to + 12.1 mV when the CMP slurry pH increased from 5.0 to 6.25, and it increased weakly from + 12.1 to + 15.3 mV for further CMP slurry pH increase, as shown Fig. 5b. This result implies that the lowest abrasive zeta potential (+ 12.11 mV) was observed at a specific pH (6.25). Additionally, the secondary abrasive size decreased considerably from 223 to 130 nm when the CMP slurry pH increased from 5.0 and 6.0, and it increased significantly from 153 to 3126 nm when the pH increased from 6.25 to 7.0. This result indicates that the minimum secondary abrasive size (130 nm), which has an excellent dispersant degree, was identified at a specific pH (6.0). As a result of the dependencies of the abrasive zeta potential and secondary abrasive size on the CMP slurry pH, the SiO_2_-film polishing rate significantly increased from 263 to 524 nm/min when the CMP slurry pH increased from 5.0 to 6.0, and then decreased considerably from 524 to 437 nm as the CMP slurry pH increased further, as shown in Fig. 5c. Additionally, the dependency of the SiO_2_-film polishing rate on the CMP slurry pH was well correlated with that of the abrasive effective contact area on the SiO_2_-film surface, that is, a higher effective contact area led to a higher SiO_2_-film polishing rate. Thus, the super-fine Ce(OH)_4_ abrasive-based CMP slurry demonstrated an excellent abrasive dispersant characteristic and a superior SiO_2_-film CMP performance (i.e., stick-and-slip type, scratch-free, and SiO_2_-film polishing rate >  ~ 500 nm/min) at a CMP slurry pH of 5.0–6.0. However, its SiO_2_-film polishing rate was sensitive to the CMP slurry pH, The SiO_2_-film polishing rate rapidly increased with the CMP slurry pH; the ratio of the SiO_2_-film polishing rate to the slurry pH was 84 nm/min per pH). In addition, the surface roughness of the SiO_2_-film surface before and after CMP were estimated by AFM, as shown in Fig. S8. The scanning area of AFM was 5 μm × 5 μm and the surface roughness was calculated by using root-mean-square (Rq). The surface roughness of the SiO_2_-film surface before CMP was 0.394-nm, while those after CMP at pH 5.00, 5.50, 5.75, 6.00, 6.25 and 6.50 were 0.360, 0.376, 0.342, 0.359, 0.366 and 0.316-nm, respectively. These result indicate that there was no degradation of the SiO_2_-film surface roughness by CMP.

## Discussion

To achieve minimal stick-and-slip type scratches and a high polishing rate with a SiO_2_-film CMP in nanoscale semiconductor devices, super-fine wet ceria abrasives were synthesized via a novel wet precipitation process using Ce^4+^ and a catalyst enhancing Ce^4+^ soluble at temperature of 25 °C. This is in contrast to the conventional wet precipitation that uses a mixture of Ce^4+^ with Ce^3+^ and an amine-based synthesized catalyst at 60–80 °C. The chemical composition of super-fine wet ceria abrasives strongly depended on the synthesis termination pH: Ce(OH)_4_ abrasives at a pH of 4.0–5.0 and a mixture of Ce(OH)_4_ with CeO_2_ at a pH of 5.5–6.5. The Ce(OH)_4_ abrasives were well dispersed in a SiO_2_-film CMP solution, and exhibited a transparent yellow color; however, the CeO_2_ abrasives, which exhibit an opaque yellow color, were significantly agglomerated in the CMP solution. As a result, Ce(OH)_4_ abrasives with excellent dispersant ability were identified at a specific synthesis termination pH (5.0), which presented the minimum positively charged abrasive zeta potential (~ 12 mV) and the minimum secondary abrasive size (~ 130 nm). Additionally, super-fine Ce(OH)_4_ wet ceria abrasives crystallized well with an f.c.c crystalline structure and an average size of ~ 2 nm. Although the abrasive size is only several nanometers, the super-fine abrasive-based SiO_2_-film CMP slurry exhibited a high SiO_2_-film polishing rate (> 500 nm/min), owing to the extremely high population of super-fine abrasives and excellent dispersion ability. Additionally, it could achieve a surface free of stick-and-slip type scratches after a SiO_2_-film CMP. It should be noted that a conventional CeO_2_ dry or wet ceria abrasive is not free of CMP-induced scratches. Furthermore, the abrasive zeta potential and secondary abrasive size in the CMP slurries, including super-fine wet ceria abrasives, were very sensitive to the CMP slurry pH, and principally determined the CMP performance, such as the SiO_2_-film polishing rate and CMP induced scratches. The best CMP performance with a minimum abrasive zeta potential (~ 13 mV) and abrasive secondary size (~ 130-nm in size) could be achieved at a specific CMP slurry pH (6.0), which was different from the synthesis termination pH (5.0). The sensitive pH characteristic of the super-fine abrasive-based SiO_2_-film CMP slurry enabled the study of the stability of the CMP slurry. Additionally, further chemical design studies to achieve a low cost (i.e., dependency of solid loading on CMP performance) and self-stop CMP function (i.e., polishing rate selectivity between SiO_2_-, Si_3_N_4_-, and poly-Si-films) are essential for various CMP applications in nanoscale semiconductor devices.

## Methods

### Ce(OH)_4_ abrasive synthesis

For super-fine wet ceria synthesis, 0.052 mol of (NH_4_)_2_Ce(NO_3_)_6_ was dissolved in DI water. The initial pH of the (NH_4_)_2_Ce(NO_3_)_3_ solution was 1.30, and the solution was dark orange in color. This indicates that (NH_4_)_2_Ce(NO_3_)_6_ is dissociated in DI water and exists as [Ce(NO_3_)_6_]^2−^ and NH^4+^. Additionally, 0.170 mol of C_3_H_4_N_2_ was dissolved in DI water to enhance Ce^4+^ solubility. The pH of the C_3_H_4_N_2_ solution was 11.4, and the solution was colorless. Then, the C_3_H_4_N_2_ solution was added to the (NH_4_)_2_Ce(NO_3_)_6_ solution at a rate of 10 ml/min for 15 min. All these synthesis processes were conducted at temperature of 25 °C and maintained at a constant stirring rate. After the C_3_H_4_N_2_ and (NH_4_)_2_Ce(NO_3_)_6_ solutions were mixed, the final pH and color of the solution were 3.01 and transparent bright yellow, respectively. Through this color change, it could be inferred that the (NH_4_)_2_Ce(NO_3_)_6_ solution is completely ionized to Ce^4+^ by comparison with the color when the C_3_H_4_N_2_ solution was not added. After synthesis, the mixture of the (NH_4_)_2_Ce(NO_3_)_6_ and C_3_H_4_N_2_ solutions was titrated to a specific pH (4.0–6.5) using NaOH as the titrant. The solution was then centrifuged three cycles at 8000 rpm.

### Materials

The CMP slurries used in this study were composed of 0.3 wt% super-fine wet ceria abrasives, a pH titrant (NaOH), a dispersant (PVA), and DI water. F.c.c. crystalline Ce(OH)_4_ was synthesized as an abrasive particle with a primary particle diameter of ~ 2 nm. Ammonium cerium (IV) nitrate ((NH_4_)_2_Ce(NO_3_)_3_, Junsei Chemical, Tokyo, Japan) was used as the precursor material for the synthesis of Ce(OH)_4_ abrasive particles. Additionally, imidazole (C_3_H_4_N_2_, Sigma Aldrich, St. Louis, Missouri, USA) was used as a catalyst to improve the solubility of (NH_4_)_2_Ce(NO_3_)_3_. Sodium hydroxide (NaOH, Junsei Chemical, Tokyo, Japan) was used to adjust the pH. PVA (Polysciences, Warrington, US) was used as a dispersant. The slurry was prepared with the following concentration: 0.3 wt% nano-scale Ce(OH)_4_ abrasives and 0.3 wt% PVA dispersant. The pH of the slurry was titrated at 6.0 using NaOH.

### CMP conditions

The 450-nm SiO_2_-film were prepared on a 12-inch-diameter Si wafer with CVD. The sample was polished using a polisher (POLI-762, G&P Tech. Inc., Republic of Korea) with an industry standard CMP pad of polyurethane-like (IC1000/Suba IV, Rohm & Haas Electronic Materials, USA). Before polishing, the polishing pad was conditioned with a diamond disk and DIW for 30 min, and then polished 3 dummy SiO_2_-film wafers. When evaluating slurries of different conditions, pad conditioning was performed for 15 min, and 3 dummy SiO_2_-film wafers were polished using DIW to remove the previous slurry from the pad. The applied polishing pressure was 3 PSI and the rotation speed of the wafer carrier and the table were set to 93 and 87 rpm/min, respectively. The flow rate of the slurry was kept at 200 mL/min, and the polishing time was set at 60 s.

### Characterization

The morphology of the abrasive particles was observed using HR-TEM (JEM-2010, JEOL, Tokyo, Japan) with an accelerating voltage of 200 kV. To investigate the dispersant degree of the SiO_2_-film CMP slurry, the absorbance of the abrasives at 292–302 nm was measured using ultraviolet-visual spectroscopy (Cary 5000, VARIAN, Palo, Alto, CA, USA). Additionally, the zeta potential and diameter of the secondary particles were analyzed using a particle analyzer (ELSZ2+, Otsuka Electronics, Osaka, Japan) with electrophoresis techniques and dynamic light scattering. For measuring the secondary abrasive diameter of a CMP slurry, the slurry was diluted until the solid loading of CMP slurry was 0.3 wt% and then the 1 ml of the diluted CMP slurry was loaded at a particles counter (ELS-Z). The thickness of the SiO_2_-film was measured using ellipsometry (V-VASE, J.A. Woollam Co., Inc., Lincoln, NE, USA). The polishing rate of the SiO_2_-film was calculated by subtracting the SiO_2_-film thickness after CMP from that before CMP. At all CMP slurries in our experimental, 3 times CMP were conducted with three 300-mm-diameter SiO_2_-film wafers and 149 points with 6.1-mm interval and 1-mm exclusion from wafer edge were measured for each of wafers. In our experiment, there were no other factors impacting the CMP SiO_2_-film polishing-rate.

## Supplementary Information

Supplementary Information.

## Data Availability

The datasets generated during and/or analyzed during the current study are available from the corresponding author on reasonable request.
