# Supplementary material for: Super fine cerium hydroxide abrasives for SiO2 film chemical mechanical planarization performing scratch free
Source: Sci Rep. 2021 Sep 6;11:17736. doi: 10.1038/s41598-021-97122-9 (PMC8421349; doi:10.1038/s41598-021-97122-9)
Supplement: Supplementary file 1 — Supplementary Information. [file 41598_2021_97122_MOESM1_ESM.pdf]

Supporting Information

Super Fine Cerium Hydroxide Abrasives for SiO_2 Film Chemical Mechanical Planarization Performing Scratch Free

*Young-Hye Son, Gi-Ppeum Jeong, Pil-Su Kim, Man-Hyup Han, Seong-Wan Hong, Jae-Young Bae, Sung-In Kim, Jin-Hyung Park, and Jea-Gun Park**

Figure S1

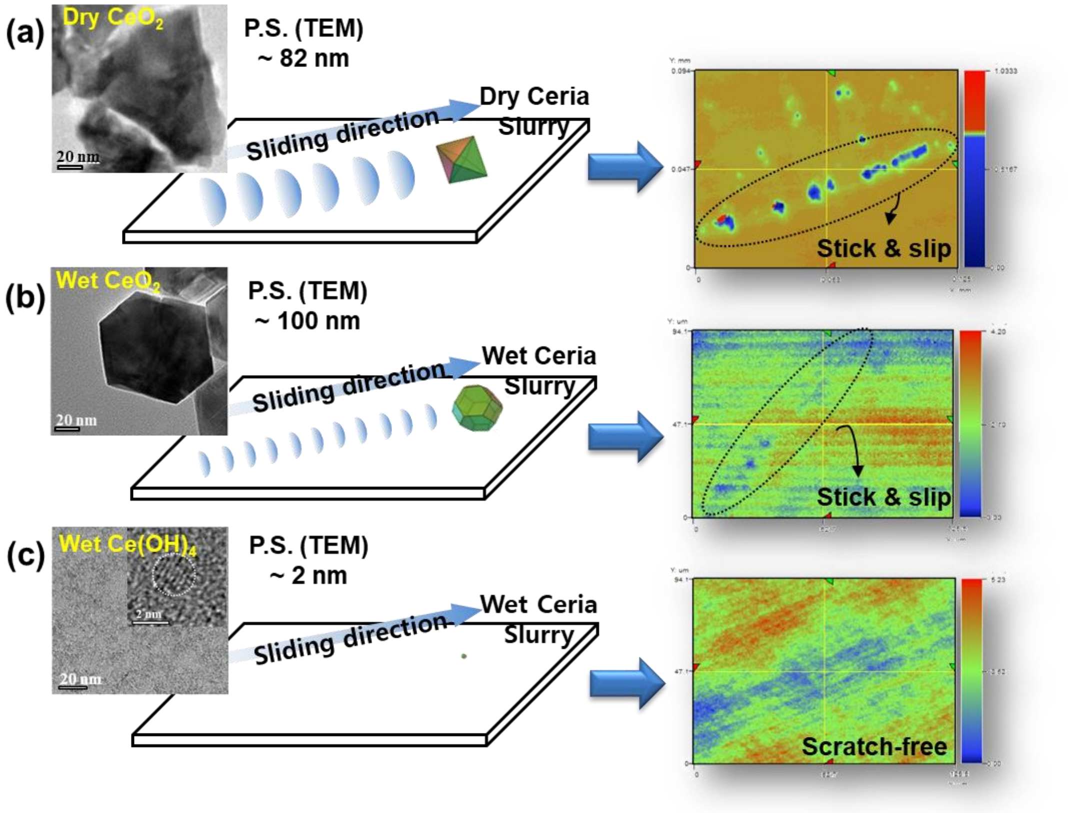

Figure S1. Schematic of generation mechanism of CMP induced scratches (i.e. slip and stick), depending on abrasive size. (a) dry CeO_2 abrasive with 82-nm in size, (b) wet CeO_2 abrasive with 100-nm in size, and (c) super-fine wet $Ce(OH)_4$ abrasive with ~2-nm in size. Dry CeO_2 abrasives are produced by a milling process of cerium carbonate, resulting in sharp and irregular surface of CeO_2 abrasive. Wet CeO_2 abrasives are synthesized by wet precipitation method using Ce^{3+}, Ce^{4+}, and NH_4OH at 60~80 ^oC , producing {111}, {110}, and {100} facets on wet CeO_2 abrasive surface. Super-fine wet $Ce(OH)_4$ abrasives are synthesized as described at our research paper.

Figure S2

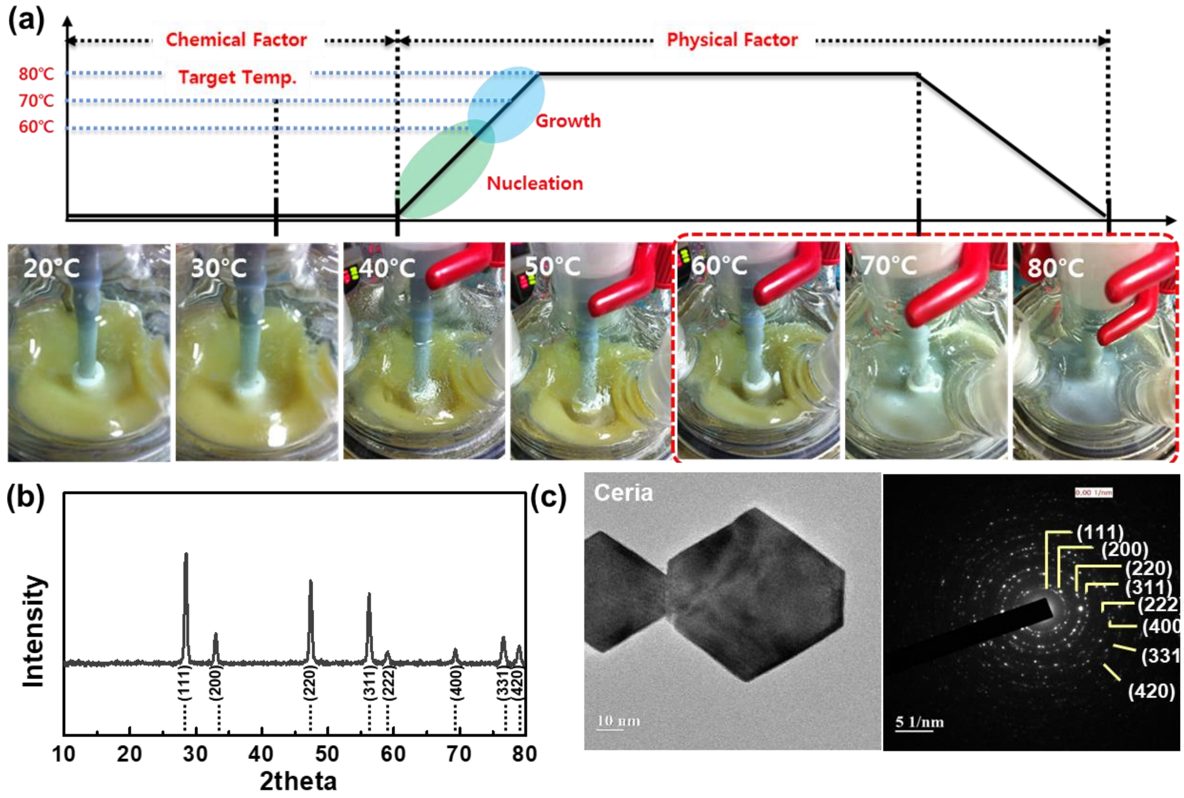

Figure S2. Morphology and crystalline properties of wet CeO_2 abrasives are synthesized using a Ce^{3+}, Ce^{4+}, and NH_4OH at 60~80 ^oC (a) temperature profile of typical wet precipitation process for synthesizing wet CeO_2 abrasives, (b) X-Ray diffraction of wet CeO_2 abrasives, and (c) high-resolution TEM image and μ-crystalline diffraction pattern. They showed a typical face-centered-cubic crystalline structure.

Figure S3

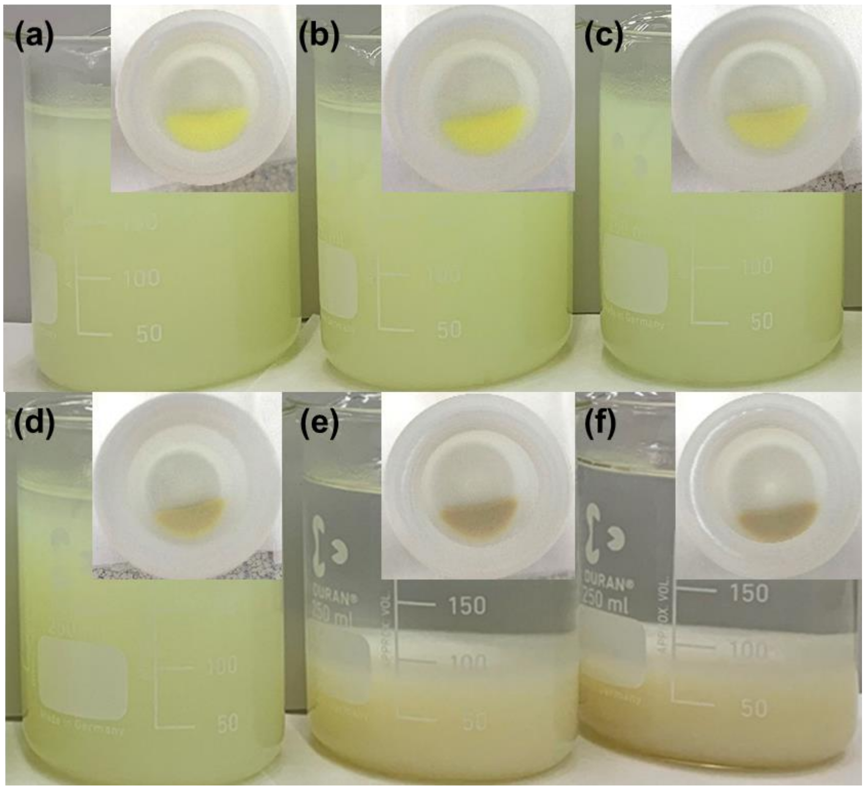

Figure S3. Dependency of colors of the solution just after synthesis termination process on synthesis termination pH. A termination pH of (a) 4.0, (b) 4.5, (c) 5.0, (d) 5.5, (e) 6.0 and (f) 6.5. The insets in figure S3 (a)-(f) demonstrated the colors of super-fine wet-ceria abrasives after 3 cycles of centrifuge. Abrasives in figures S3 (a)-(c) showed a typical color of $Ce(OH)_4$ abrasives, while those in figures S3 (d) and (e) exhibited a mixture color of $Ce(OH)_4$ with CeO_2 abrasives. Abrasive in figure S3 (f) presented a typical color of CeO_2.

Figure S4

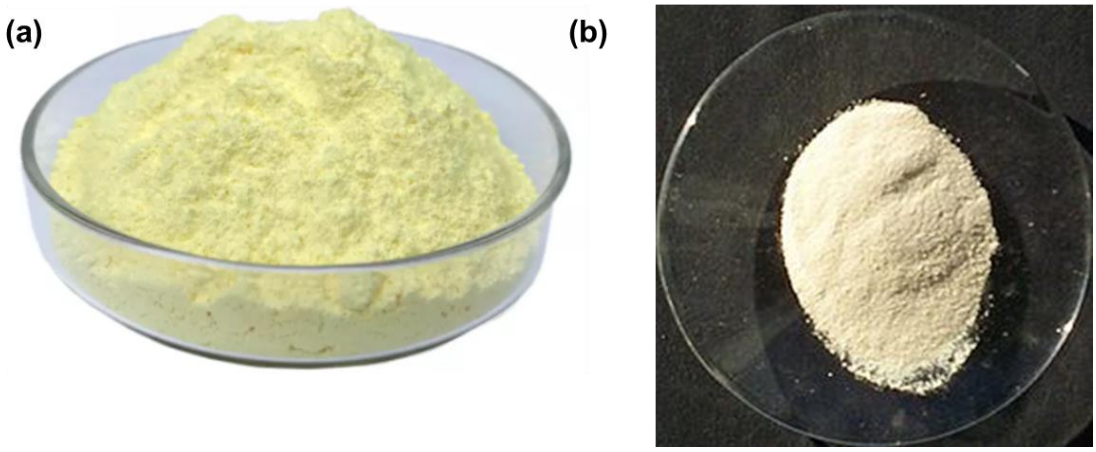

Figure S4. Abrasive colors of (a) $Ce(OH)_4$ powders[1] and (b) CeO_2 powders[2].

Figure S5

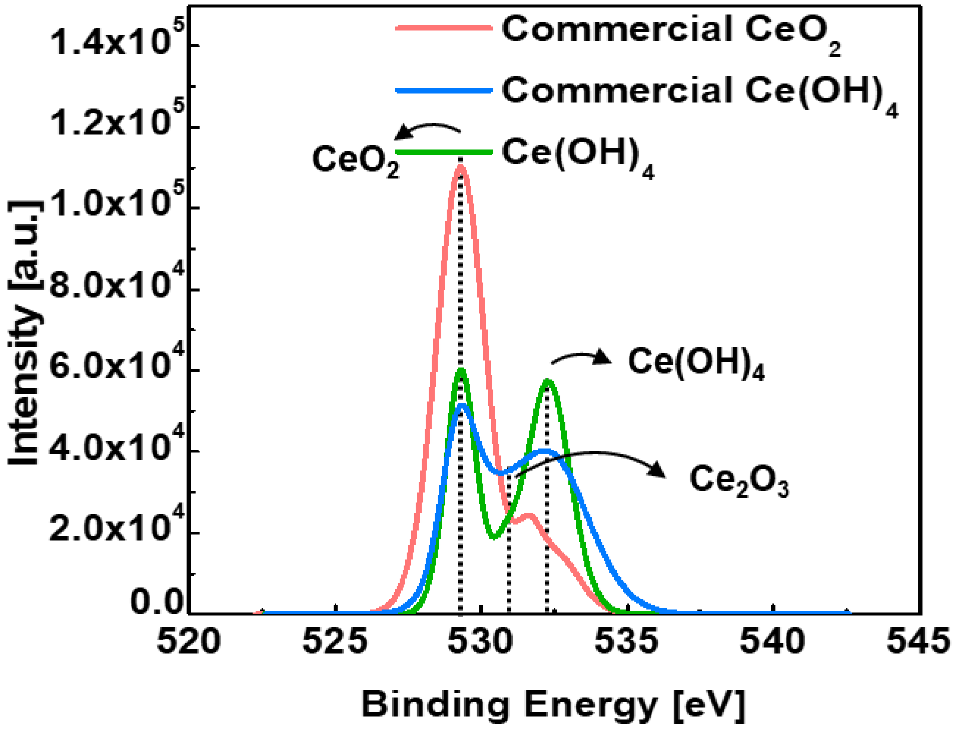

#	Ceria	1Os	CeO_2	Ce_2O_3	$Ce(OH)_4$
1	CeO_2 (Commercial)	Binding energy [eV]	529.29	530.79	532.29
		Relative area [%]	83.94	5.74	10.32
2	$Ce(OH)_4$ abrasive (HYU synthesis)	Binding energy [eV]	529.29	530.79	532.29
		Relative area [%]	39.47	6.83	53.70
3	$Ce(OH)_4$ (Commercial)	Binding energy [eV]	529.29	530.79	532.29
		Relative area [%]	21.12	26.46	52.42

Figure S5. Chemical composition analysis via XPS. Relative XPS intensity vs. binding energy for commercial CeO_2 (UB materials Co.), commercial $Ce(OH)_4$ (Sigma Aldrich Co.), and $Ce(OH)_4$ (our HYU synthesis) abrasives. The below Table represented a relative XPS peak intensity at CeO_2, Ce_2O_3, and $Ce(OH)_4$, as a function of abrasives types.

Figure S6

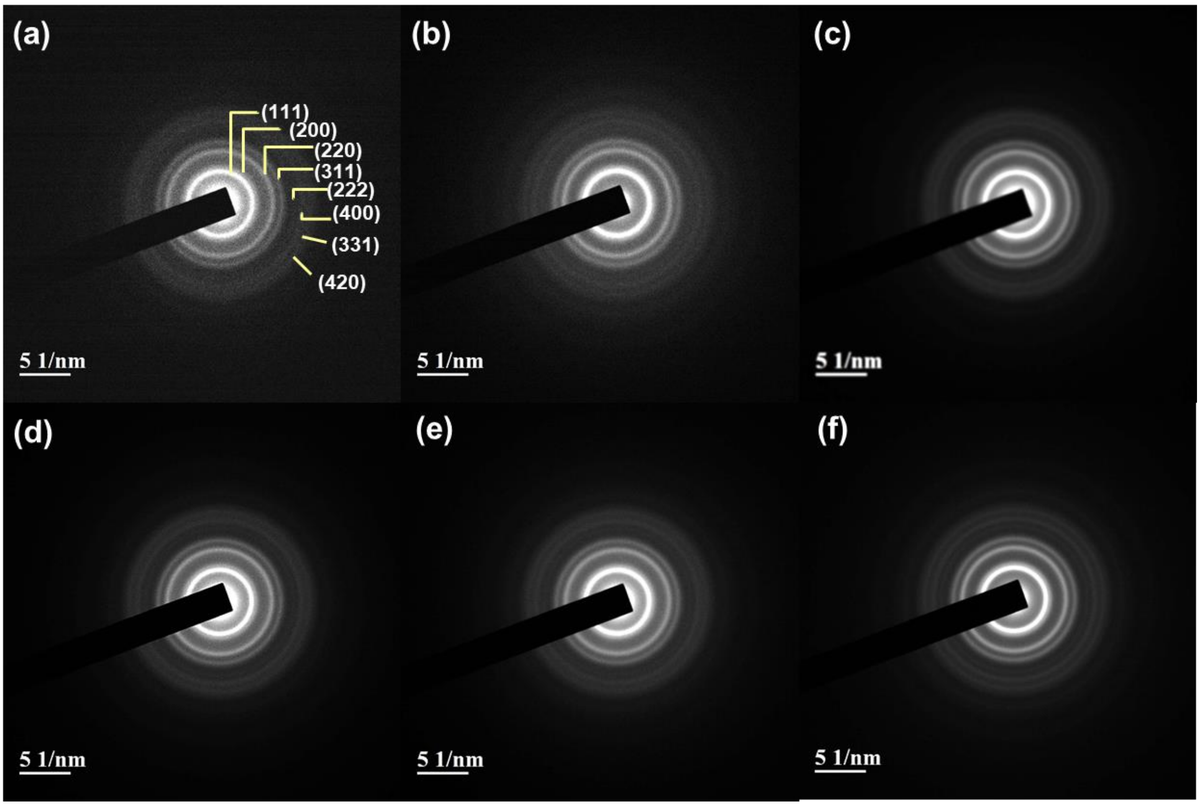

Figure S6**.** μ-crystalline diffraction patterns of super-fine wet-ceria abrasives dependent on a synthesis termination pH value of (a) 4.0, (b) 4.5, (c) 5.0, (d) 5.5, (e) 6.0, and (f) 6.5. All patterns showed a typical polycrystalline face-centered-cubic structure.

Figure S7

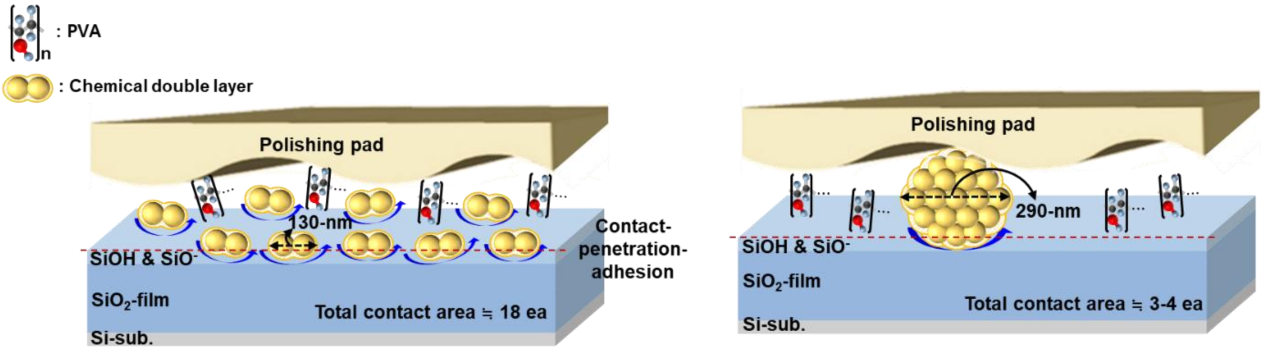

(a) (b)

Figure S7. Mechanism of contact-area based SiO_2-film CMP. Schematic SiO_2-film CMP using the slurry with (a) small secondary-abrasive size and (b) large secondary-abrasive size.

Figure S8

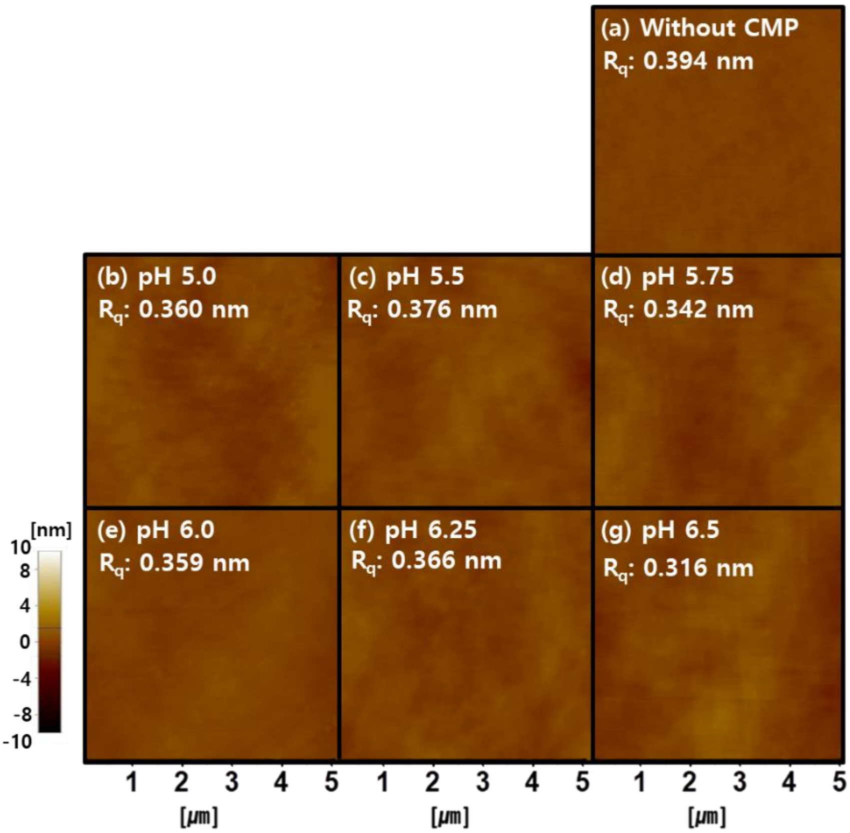

Figure S8. Influence of CMP on SiO_2-film surface roughness. (a) without CMP, (b) CMP at pH 5.00, (c)5.50, (d) 5.75, (e) 6.00, (f) 6.25, and (g) 6.50.

Reference

[1] Sichuan Wonaixi New Material Technology Co., Ltd. http://www.wnxmaterials.com/Cerium-hydroxide-pd42322335.html

[2] Walkerma. A sample of cerium oxide (CeO_2), August 2005 ; 24 August 2005, https://en.wikipedia.org/wiki/Cerium_oxide
